# Fracture Toughness, Breakthrough Morphology, Microstructural Analysis of the T2 Copper-45 Steel Welded Joints

**DOI:** 10.3390/ma13020488

**Published:** 2020-01-20

**Authors:** Hao Ding, Qi Huang, Peng Liu, Yumei Bao, Guozhong Chai

**Affiliations:** 1 Key Laboratory of E&M, Ministry of Education & Zhejiang Province, Zhejiang University of Technology, Hangzhou 310014, China; dinghao@zjut.edu.cn (H.D.); huangqijob@sina.com (Q.H.); liu_394962328@163.com (P.L.); 2 School of Mechanical Engineering, Zhijiang College of Zhejiang University of Technology, Shaoxing 312030, China; baoym@zjut.edu.cn

**Keywords:** dissimilar welding materials, electron-beam welding, fracture morphology, fracture toughness, crack deflection, three-point bending test

## Abstract

The performance and flaws of welded joints are important features that characteristics of the welding material influence. There is significant research activity on the performance and characteristics of welding joint materials. However, the properties of dissimilar welding materials and the cracking problem have not been thoroughly investigated. This investigation focuses on the evaluation and analysis of fracture mechanics, including fracture toughness, microstructural analysis, and crack initiation of T2 copper-45 steel dissimilar welding materials. Standard tensile and three-point bending experiments were performed to calculate the ultimate strength, yield strength, and elastic modulus for fracture toughness. The macro/micro-fracture morphology for tensile fracture and three-point bending fracture were analysed. Based on these investigations, it was concluded that the fracture types were quasi-cleavage and an intergranular brittle fracture mixed model. The deflection of the crack path was discussed and it was determined that the crack was extended along the weld area and tilted towards the T2 copper. Finally, the crack propagation and deflecting direction after the three-point bending test could provide the basis for improvement in the performance of welded joints based on experimental testing parameters and ABAQUS finite element analysis.

## 1. Introduction

The welding of dissimilar metals has been an area of active investigations for many years. This objective reflects an overall industrial need of increasing importance that is predicated on the technical and economic potential of the process [1]. Dissimilar metals are welded to achieve physical flexibility, but this practice often results in problems that negatively affect the performance of the weld [2]. Many researchers have investigated the effects of the welding method used for different materials that are characterized by different electrochemical [3], thermal [4], optical [5], and mechanical properties [6,7,8,9], especially dissimilar metals [9,10,11,12,13,14,15,16,17,18,19,20,21]. In general, for conventional joints with two dissimilar metals, the primary concern is the potential effect of the unique properties of the materials on the fusing process and further determines the mechanical behavior of the joint [22]. It has been determined that welding defects are highly related to mechanical properties. In the case of keyhole pores, the formation is controlled by the temperature gradient and surface tensions of the liquid/solid interface [23], when the selective laser melting (SLM) defects quantity increase to a certain proportion, the tensile strength, fatigue life, and hardness of the dissimilar joint are dramatically affected [24].

Generally, the physical and chemical properties of copper and carbon steel are quite different. The thermal conductivity of copper is 7–11 times that of steel and the melting point is 400–500 degrees Celsius lower than steel. However, at high temperatures, the atomic radius, lattice types, and lattice constants of Fe and Cu are very close. These similarities are beneficial in the welding of copper and steel dissimilar materials [15]. T2 copper is a commonly used metal material in industry. It is often used as the material of large container structure because of its high chemical stability and good corrosion resistance in a calcium salt environment [25]. However, T2 copper has low strength and a large specific gravity, which results in its limitation in lightweight design [11]. In contrast, high strength and easy cutting behavior characterize 45 Steel. These contrasting properties imply that the resulting joints due to these two metals would have broad application prospects. The fusion zone (FZ) microstructures in the electron beam welding of copper-stainless steel were investigated and the results indicated the existence of some defects, such as porosity and micro-fissures, which are mainly influenced by the process and geometry parameters [2]. More interestingly, by appropriately adjusting the welding parameters during electron beam welding, the porosity and micro-cracks can be effectively reduced in the heat-affected zone (HAZ) and FZ; this was strongly controlled by a high-temperature gradient [25]. The high-temperature gradient in the electron beam welding process compensated for the influence of the temperature difference between copper and steel on the solid-liquid interface and FZ [3].

Many researchers have investigated the mechanical behavior of dissimilar welding joints; however, most of the studies have focused on the effect of welding defects on the tensile strength of dissimilar welding materials [1]. Analysis of the fracture characteristics using finite element simulation has seldom been performed, while the control of microstructure during various welding processes has been well investigated [26]. The effect of an intermetallic compound on mechanical properties has also been well-studied, but the available information on fracture performance is still limited [27]. The formation of intermetallic phases greatly affects the interfacial strength of dissimilar welding materials as a result of the different melting temperatures, particularly for copper-steel dissimilar welding materials [2]. The crack propagation mechanism of dissimilar welding materials has generated general interest, and some reports have demonstrated the mechanism of surface crack propagation of these materials by combining microstructures e.g., ferrite and austenite [27,28]. Moreover, many investigators have analysed the effect of crack position on the fracture behaviour that is based on the three-point bending tests [29]. A few researchers have summarized the effect of some regular patterns on crack propagation [30]. Crack ductility fracture occurs in low-strength materials and the distance between the crack initiation point and interface affect the fracture behavior [31]. The numerical simulation of crack propagation was consistent with experimental results. The extended finite element method (XFEM) [32,33] is frequently utilized to simulate crack propagation [34]. XFEM has also been applied to simulate the crack propagation of contact fatigue [35], which was analysed based on two-dimensional and three-dimensional contact fatigue tests [36,37]. Various models of crack propagation have been established to be consistent with actual situations [38].

At present, research on the welding materials of T2 copper and 45 steel mainly focuses on the influence of the welding solder on the interfacial strength. However, correlative research on electron beam welding is limited. As such, it is important to study the joint property and fracture behaviour of the electron beam welding specimens.

In this work, a copper/steel dissimilar welding specimen was prepared by electron beam welding (without filler wire) to examine the weld properties of these joints. The microstructure of the copper/steel dissimilar welding materials of the weld area was analysed via the combination of micro-topography and macroscopic appearance. Accordingly, the relationship between crack deflection was demonstrated based on the material properties. Moreover, the fracture mechanics test parameter was examined on the basis of tensile properties and bending experiments.

## 2. Materials and Methods

### 2.1. Parameter Test Method for Joint Property

The test materials were prepared while using commercial welding processes and electron beam welding equipment. The model of electron beam welding machine was SEBW and the manufacturer was Guilin Shichuang vacuum CNC Equipment Co., Ltd. (Guilin, China). After investigation, Table 1 shows the parameter cases of some scholars in the electron beam welding of copper-steel.

**Figure 1:**
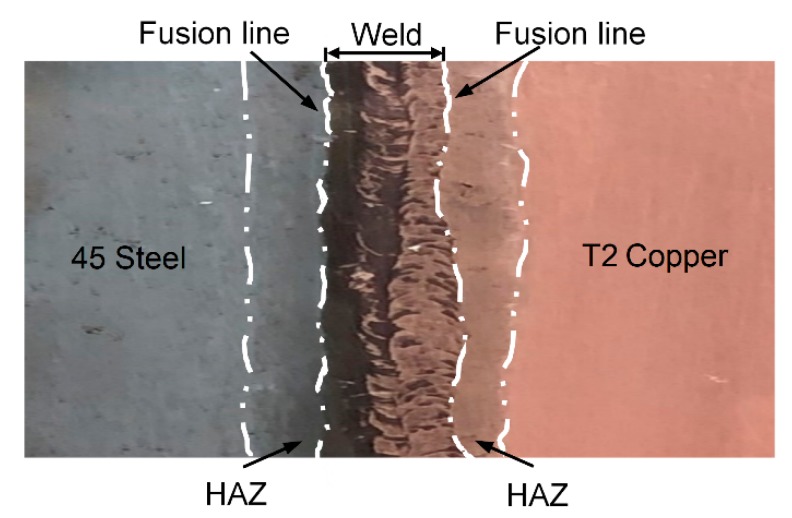
Welded sample.

We preferentially adjust the parameters with reference to Table 1. After actual testing, the electron beam welding parameters of T2 Copper/45 steel dissimilar welding materials were as follows: acceleration voltage 80 kV, electron beam 100 mA, vacuum degree 5 × 10^−2^ torr, and welding speed 300 mm/min. The surface of the welding sample and the HAZ had a visible dividing line with the weld, which is clearly shown in Figure 1. Table 2 shows the chemical composition of T2 copper and 45 steel.

**Figure 2:**
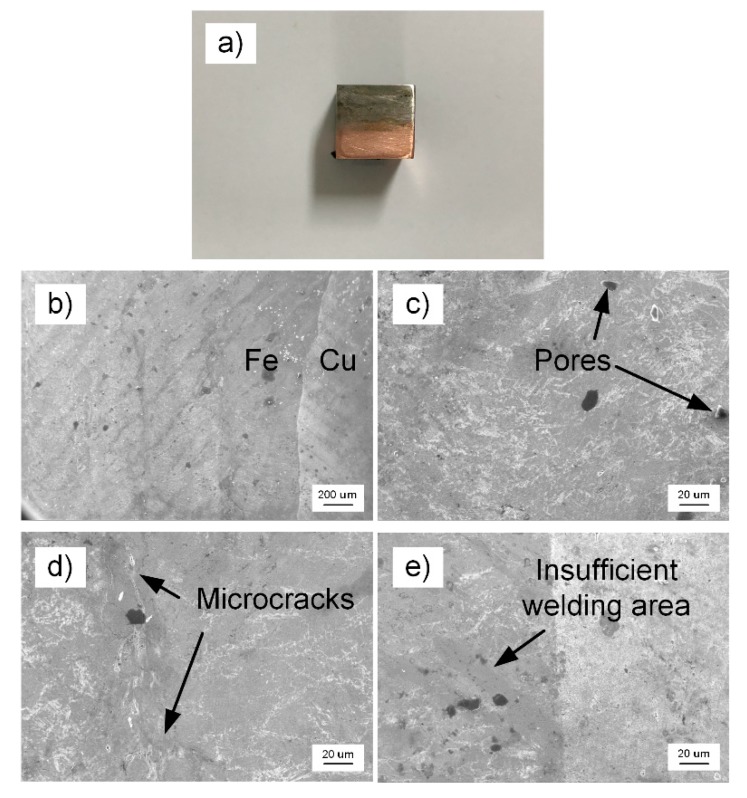
Scanning electron microscopy (SEM) micro-morphology of welded area (**a**) square sample by Electrical Discharge Machining (EDM); (**b**) magnification is 50 times; and, (**c**,**d**,**e**) magnification is 500 times.

A 4 × 8 × 10 mm square sample was taken from the welded sample by Electrical Discharge Machining (EDM), as shown in Figure 2a, and Figure 2b–e show the microscopic topography of welded area via scanning electron microscopy (SEM) after polishing. The magnifications were 50 and 500 times, respectively. Figure 2b displays the iron and copper ends of the weld area. In Figure 2c–e, some pores, microcracks, and the insufficient welding area can be seen. These defects are the important factors that affect the welded bond quality of T2 copper-45 steel.

**Figure 3:**
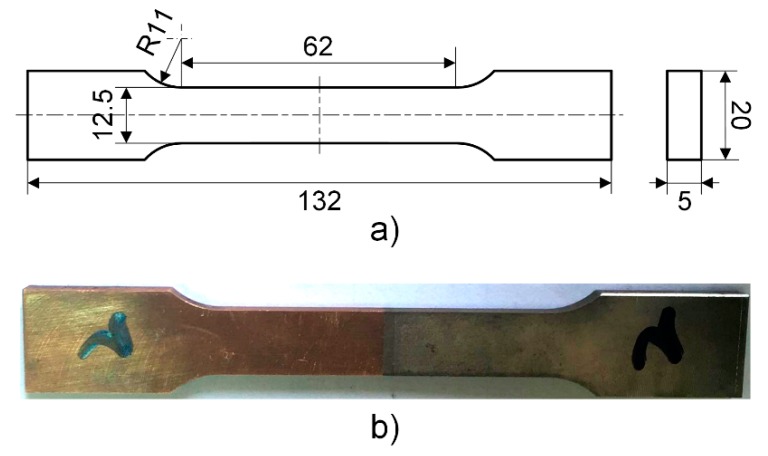
Tensile specimen (**a**) dimension (mm); and, (**b**) actual sample.

The tensile specimen was obtained by wire cutting according to GB/T228.1-2010 [46] (Metallic Materials-Tensile Testing-Part 1: Method of testing at room temperature). The ultimate strength, yield strength, and elastic modulus were determined while using the INSRON-8801 Servohydraulic Fatigue Testing System (Instron, Darmstadt, Germany) with a loading rate of 1 mm/min. Figure 3 shows the tensile specimen.

**Figure 4:**
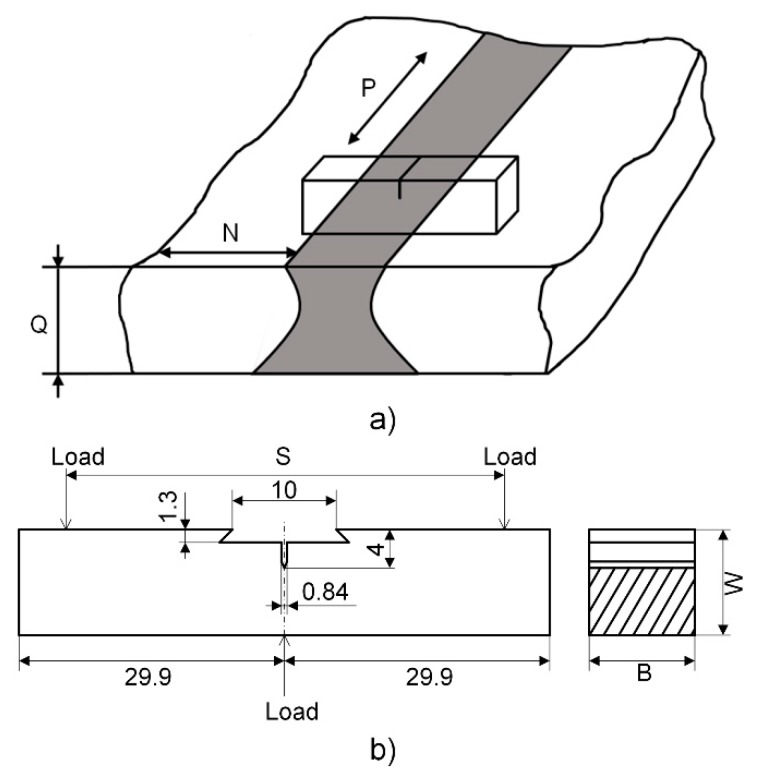
Three-point bending (**a**) specimen sampling orientation; and, (**b**) dimension (mm).

The processing of the three-point bending specimen was based on GB/T21143-2014 [30] (unified method of test for determination of quasi-static fracture toughness) and GB/T 28896-2012 [47] (metallic materials-method of test for the determination of quasi-static fracture toughness of welds). The sampling orientation of the fracture surface of the fracture toughness specimen in the weld zone was NQ, as shown in Figure 4a, the maximum fatigue preformed twill force was set according to the smaller value of Equations (1) and (2), the maximum fatigue crack stress was calculated as *F_f_* is 1344.7169 N at the last 1.3 mm or 50% fatigue precracking propagation, and the stress ratio *r* is 0.5.

(1) $$F_{f}=0.8\times \frac{B{\left(W-a_{0}\right)}^{2}}{S}\times R_{p_{0.2}}$$

(2) $$F_{f}=\xi \cdot E\left[\frac{{\left(W\cdot B\cdot B_{N}\right)}^{0.5}}{g_{1}\left(\frac{a_{0}}{W}\right)}\right]\cdot \left(\frac{W}{S}\right)$$

In the preceding Equations (1) and (2), the dimensional coefficient *ξ* is 1.6 × 10^−4^ m^1/2^, *B* is the sample thickness that is shown in Figure 4b, *W* is the width of the specimen, *B_N_* is the net thickness of the specimen and *B*, *B_N_*, *W* are 13 mm; the span *S* is 52 mm, the initial crack length *a*_0_ is 6 mm, the stress intensity factor coefficient *g*(*a*_0_/*W*) is 2.29; *E* is the elastic modulus; and, *R_p_*_0.2_ is the specified plastic elongation strength of the material in the vertical crack plane 0.2% at the test temperature.

**Figure 5:**
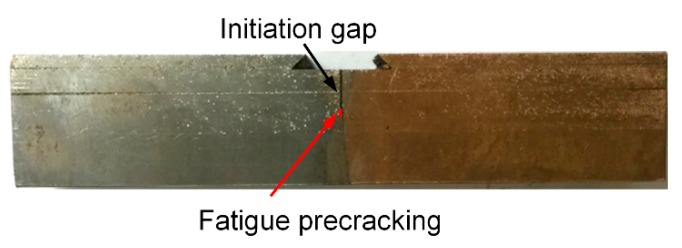
Final specimen of three-point bending.

The fatigue crack was prepared while using the constant load method. After this process, the fatigue precracking of the three specimens was: 2.02, 1.96, and 2.04 mm. Figure 5 shows the final specimen of three-point bending.

### 2.2. Characterization Results of Joint Property Parameters

**Figure 6:**
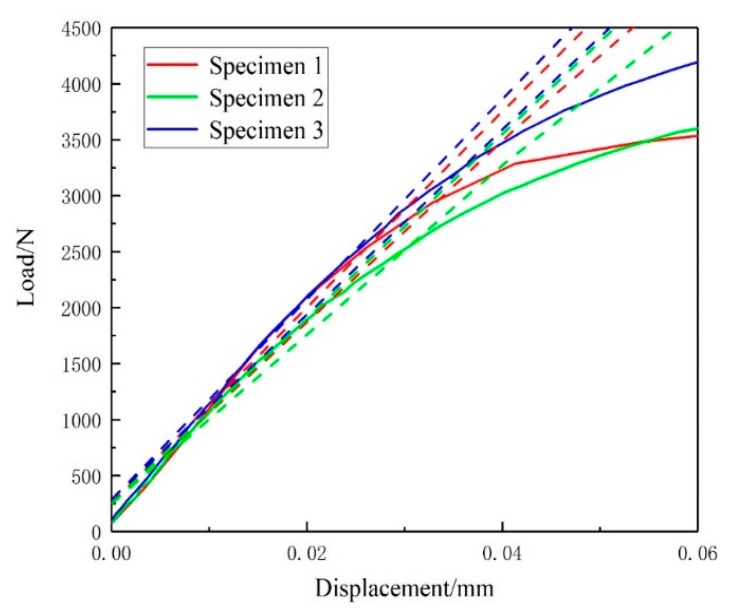
Displacement-force (P-V) curve of three-point bending.

Table 3 shows the performance parameters were obtained by standard tensile tests and the results. The displacement-force curve (P-V curve) of the notch opening was obtained based on the three-point bending test that is shown in Figure 6.

After the P-V curve of the three-point bending specimen was shifted, the value *F_Q_* of the three specimens was 3286.569, 2727.193, and 3581.864 N.

The judgment basis is as follows.

(3) $$\frac{F_{\max}}{F_{Q}}\geq 1.1$$

where *F_Q_* is the maximum force and *F*_max_ is the maximum force that the specimen can withstand.

Given that *F*_max_/*F_Q_*_1_, *F*_max_*/F_Q_*_2_, and *F*_max_/*F_Q_*_3_ are greater than 1.1, *K*_max_ (conditional value of *K_IC_*) was calculated while using Equation (4).

(4) $$K_{\max}=K_{Q}=\left[\left(\frac{S}{W}\right)\frac{F_{Q}}{{\left(B\cdot B_{N}\cdot W\right)}^{0.5}}\right]\cdot \left[g_{1}\left(\frac{a_{0}}{W}\right)\right]$$

The judgment on plane strain fracture toughness *K_IC_* is represented, as follows.

(5) $$a_{0}=2.5{\left(\frac{K_{Q}}{R_{p_{0.2}}}\right)}^{2}$$

(6) $$\left(W-a_{0}\right)=2.5{\left(\frac{K_{Q}}{R_{p_{0.2}}}\right)}^{2}$$

(7) $$B=2.5\left(\frac{K_{Q}}{R_{p_{0.2}}}\right)$$

(8) $$K_{f}=0.6K_{Q}\left(\frac{{\left(R_{p_{0.2}}\right)}_{p}}{{\left(R_{p_{0.2}}\right)}_{e}}\right)$$

where *K_Q_* can be acquired from the three-point bending test, (*R_p_*_0.2_)*_e_* is the plastic extension strength corresponding to the bias 0.2% at the test temperature, and (*R_p_*_0.2_)*_p_* is the plastic elongation corresponding to the fatigue precracking offset 0.2%.

After the aforementioned judgement, *a*_0_ is the initial crack length, *W* − *a*_0_ is the difference between the sample width and the initial crack, and *K_f_* is the maximum value of the stress intensity factor in the final stage of the prepared fatigue crack. Table 4 presents these parameters.

(9) $$K_{IC}=\frac{K_{Q1}+K_{Q2}+K_{Q3}}{3}=6.027\mathrm{MPa}\cdot m^{1/2}$$

According to results from the data that are contained in Table 4 and Equation (9), the fracture toughness of the welded joint calculated in the three-point bending test is 6.027 MP·m^1/2^.

## 3. Results and Discussion

### 3.1. Fracture Analysis of Tensile Test

**Figure 7:**
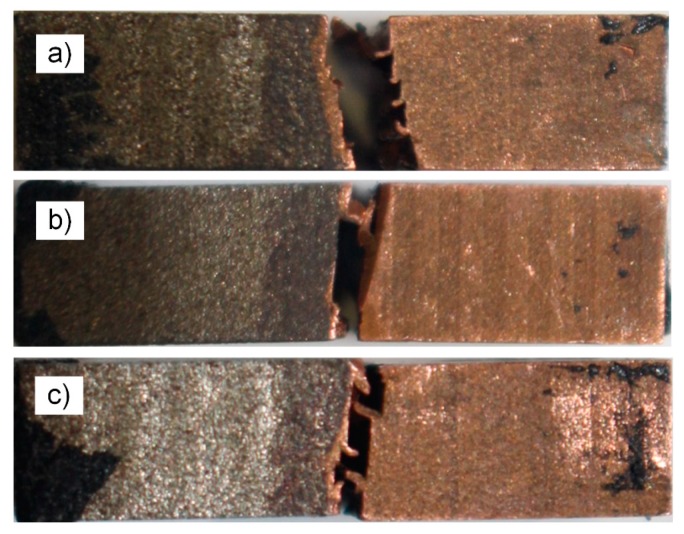
Macroscopic tensile fracture (**a**) for specimen 1; (**b**) for specimen 2; and, (**c**) for specimen 3; steel on the left and copper on the right.

**Figure 8:**
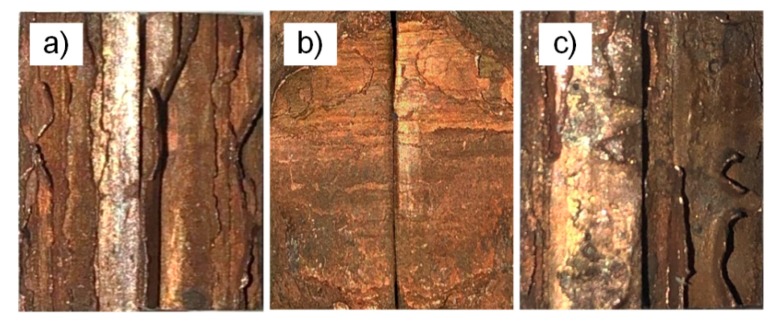
Macro-morphology fracture surface of tensile specimen (**a**) for specimen 1; (**b**) for specimen 2; and, (**c**) for specimen 3; steel on the left and copper on the right.

After tensile testing, the macroscopic fracture area of the specimen is shown in Figure 7 and Figure 8. The specimen breaks in the weld zone and crack propagation was biased towards the copper interface. The copper can be observed on the fracture surface, which was partially attached to the ends.

**Figure 9:**
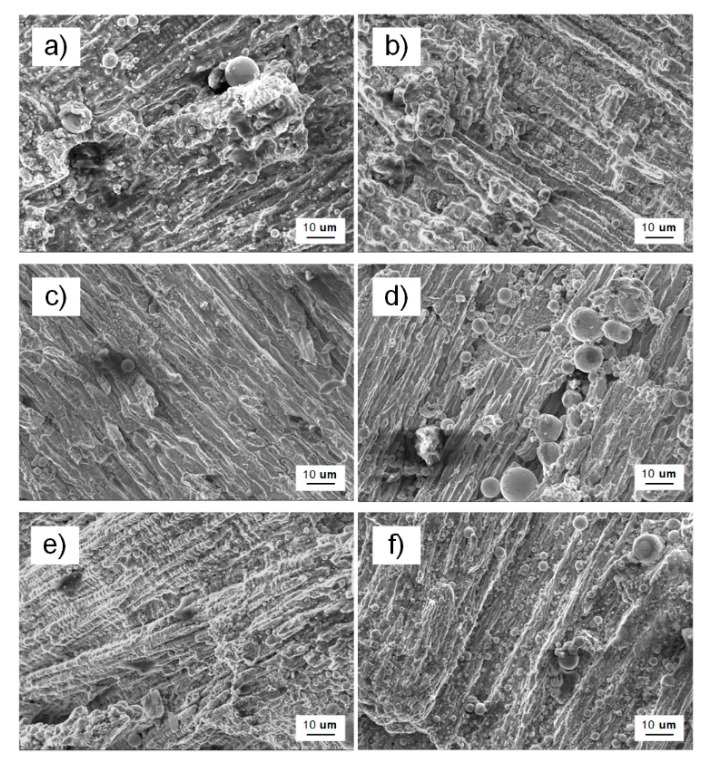
SEM micro-morphology fracture surface of tensile specimen (**a**,**b**) for specimen 1; (**c**,**d**) for specimen 2; and, (**e**,**f**) for specimen 3; steel on the left and copper on the right.

The microscopic topography of the tensile fracture of the T2 copper/45 steel dissimilar welding materials is shown in Figure 9 via SEM at a magnification of 1000 times for each specimen two-fracture end face, according to the order of the macroscopic fracture morphology in the immediately preceding figures.

The macroscopic shape of specimen 1 had obvious gloss and irregular geometry, and the shiny surface of the fracture was almost perpendicular to the normal stress, which is associated with the brittle fracture characteristic; and, significant grain-brittle fracture characteristics at the microscopic level. There was a network structure after fracture due to an external force, which was a relatively obvious network brittle phase, as shown in Figure 9b. The reason for this fracture was the brittle precipitation phase on the grain boundary, which results in the formation of a continuous carbide network by allotropes of iron during electron beam welding, which led to a thin layer of brittle fracture splitting.

Brittle fracture also characterized the macroscopic fracture of specimen 2, which appeared as herringbone and radial patterns at the fracture with a shiny surface. There were fluvial, blocky, and spherical structures in the microscopic topography with cleavage steps and tearing ribs, which exhibited the microscopic features of crystal brittleness and cleavage fracture [41,48].

A few flaky smooth surfaces existed in the macroscopic fracture of specimen 3 and the entire fracture surface was relatively flat. The cleavage characteristics of trapezoidal and river patterns also appeared in the microscopic morphology, with tiny cleavage steps and tearing ribs that are associated with the cleavage fracture. The defects in the weld area and the impurities of the welding material caused this microscopic appearance.

### 3.2. Fracture Analysis of Three-Point Bending Test

**Figure 10:**
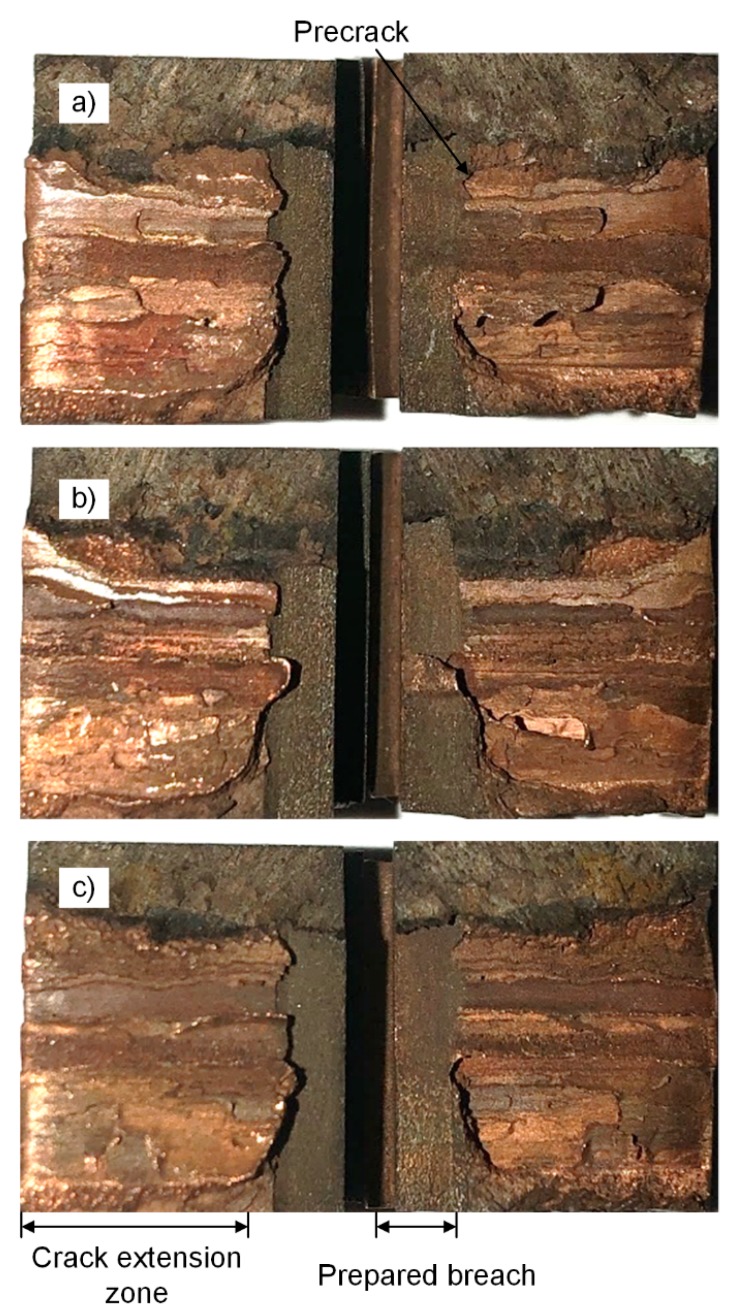
Macro-morphology fracture surface of three-point bending specimen (**a**) for specimen 1; (**b**) for specimen 2; and (**c**) for specimen 3; steel on the left and copper on the right.

The macroscopic fracture surface is shown in Figure 10 after the three-point bending test and the prepared breach prepared fatigue crack and crack extension zone can be observed.

**Figure 11:**
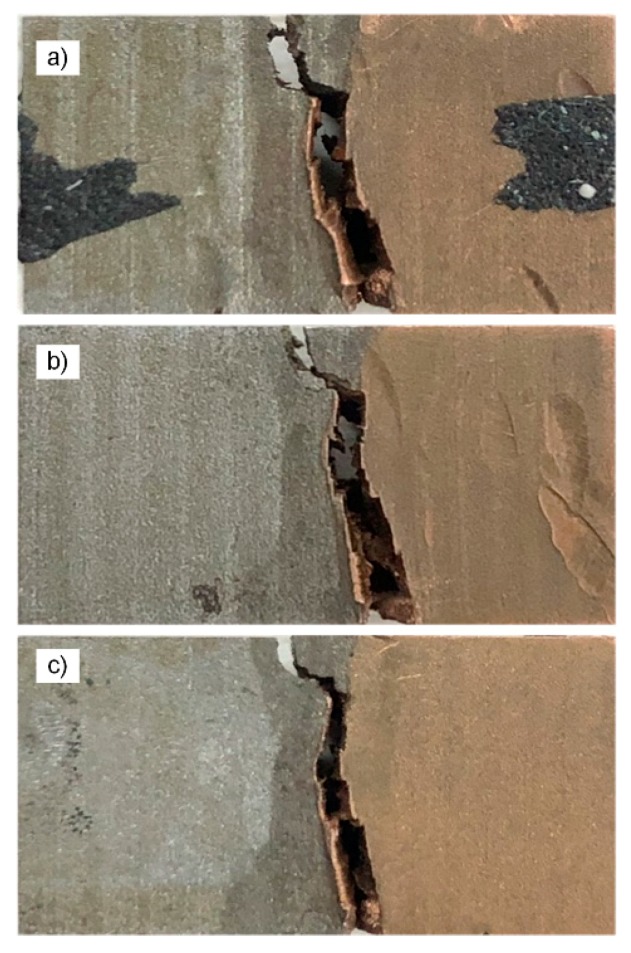
Macroscopic crack propagation of three-point bending specimen (**a**) for specimen 1; (**b**) for specimen 2; and (**c**) for specimen 3; steel on the left and copper on the right.

The purplish-red hue gradually deepens from the top to the bottom in the crack extension zone and the copper attached to the fracture surface gradually increased. It was known that the crack gradually deflected along the copper thereby tearing copper that was attached to the surface, as shown in Figure 11.

**Figure 12:**
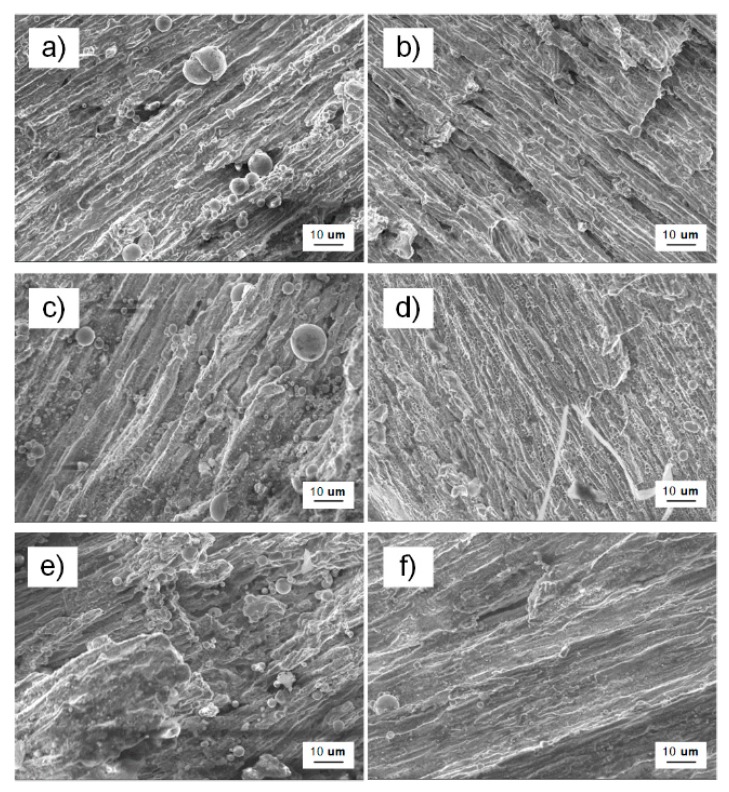
SEM micro-morphology fracture surface of three-point bending specimen (**a**,**b**) for specimen 1; (**c**,**d**) for specimen 2; and, (**e**,**f**) for specimen 3; steel on the left and copper on the right.

The three-point bending fracture was observed at 1000 times magnification while using SEM. As shown in Figure 12, these fractures in the macroscopic image have an obvious shiny surface and irregular geometry. The fluvial, blocky, and spherical structures were readily apparent in the microscopic topography with the cleavage steps and tearing ribs distributed, therefore, it was a typically mixed mode of brittle intergranular and quasi-cleavage fracture.

### 3.3. Analysis of Crack Propagation Direction

**Figure 13:**
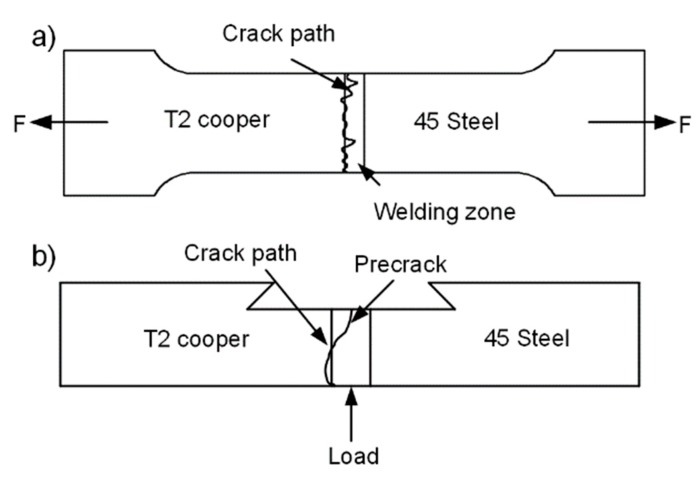
Crack deflection diagram (**a**) Standard tensile test; and (**b**) Three-point bending test.

The crack propagation path deflection of the dissimilar metal welding materials always deflects to the low strength material region [19]. The attached copper on the fracture area was caused by the deflection tear of the fracture path based on the aforementioned tensile test fracture morphology. Figure 13a shows a schematic diagram of the crack deflection of the standard tensile specimen, which was similar to the three-point bending test of T2 copper/45 steel dissimilar welding materials deflection path. This resulted in the phenomenon of stepwise reduction of the resistance to fracture due to the difference in the toughness between the weld area, HAZ and base metal in the electron beam welding process. Based on the three-point bending test of T2 copper/45 steel dissimilar welding materials cracking failure, the crack path was deflected due to the difference in the toughness, subject to factors, such as pores, micro-cracks in the weld area, crack deflection to T2 copper, as shown in the schematic diagram in Figure 13b.

Therefore, the crack propagation path of T2 copper/45 steel dissimilar welding materials always deflected to the low strength side of the T2 copper because the strength mismatch between three regions was comparatively large and the toughness decreases from the weld area to HAZ and then the base metal.

**Figure 14:**
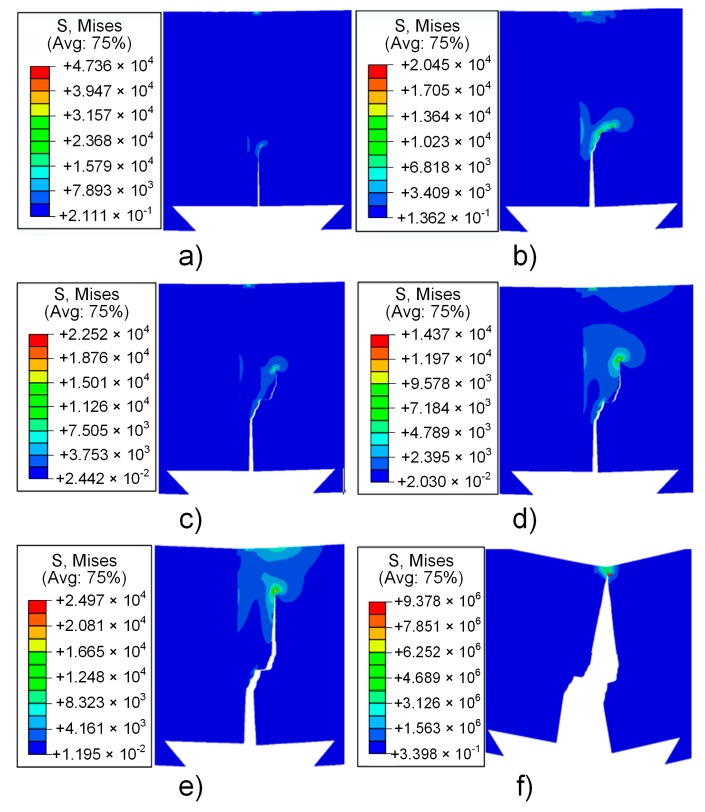
Three-point bending crack propagation simulation (**a**) step 1; (**b**) step 2; (**c**) step 3; (**d**) step 4; (**e**) step 5; (**f**) step 6.

According to the test parameters and conditions, the simulation of crack propagation of the three-point bending test was performed by ABAQUS, and the results are shown in Figure 14. With the increase of the expansion step, the crack expanded along the weld seam position and it was initially biased toward the T2 copper. The crack expanded along the junction until the specimen broke when the crack extended to the junction of the weld seam area and T2 copper. It is clear that the ABAQUS simulation results are consistent with these observations, as represented in Figure 11.

## 4. Conclusions

For the T2 copper-45 steel dissimilar welding materials that were made by electron beam welding, the joint strength, microstructural analysis, and crack initiation were explored. Based on the standard tensile test, the ultimate strength of T2 copper/45 steel dissimilar welding materials were determined to be 93.73 MPa, the yield strength was 75.37 MPa, and the elastic modulus was 108.86 GPa. It can be seen that the mechanical properties of the weld area are significantly different from those of copper and steel, which causes the strength mismatch between three regions. Through the three-point bending test, the fracture toughness was determined to be 6.027 MPa·m^1/2^, which was lower than that of pure copper (approximately 8 MPa·m^1/2^–10 MPa·m^1/2^) [49]. This is due to welding defects in the weld area. Some pores and microcracks were found in SEM micro-morphology of the welded area, which directly leads to the reduction of the mechanical properties. Weld defects indicate that, in practical application, the electron beam welding process needs to be optimized, or more suitable welding methods need to be found.

The SEM micro-morphology fracture surface of three-point bending specimen shows that the fracture type was a mixed mode of brittle intergranular and quasi-cleavage fracture. The observation results of macroscopic crack propagation of three-point bending specimen were consistent with the theoretical and ABAQUS analysis, it was concluded that the cracking path was extended along the weld area and biased towards the T2 copper. Moreover, the strength of mismatch and toughness reduction controlled the deflection.

## Figures and Tables

**Table 1:** Several cases of electron beam welding parameters of copper-steel.

Case	Thickness /mm	Acceleration Voltage/kV	Electron Beam/mA	Welding Speed/mm·min^−1^
Kar, J. [25,39]	3	60	65, 73, 80	1000
Guo, S. [40]	5	60	43–70	600
Zhang, B.G. [15,41]	2.7	60	25, 30, 35	100, 200, 300
Chen, G. [42]	5	60	15	400
Tomashchuk, I. [43]	2	20–40	20–40	200–900

**Table 2:** Chemical composition of T2 copper [44] and 45 steel [45].

**Sample**	**Cu + Ag (Minimum Value)**		**Bi**	**Sb**	**As**	**Fe**	**Pb**	**S**
T2 copper	99.90		0.001	0.002	0.002	0.005	0.005	0.005
**Sample**	**C**	**Si**	**Mn**	**P**	**S**	**Cr**	**Ni**	**Cu**
45 steel	0.42–0.50	0.17–0.37	0.50–0.80	0.035	0.035	0.25	0.30	0.25

**Table 3:** Tensile test results.

No.	Ultimate Strength	Yield Strength	Elastic Modulus
	*σ_b_*/MPa	*σ_s_*/MPa	*E*/GPa
1	102.45	81.02	88.37
2	84.67	78.64	110.53
3	94.06	66.45	127.68
Average value	93.73	75.37	108.86

**Table 4:** *K_IC_* data from calculation.

No.	*K_Q_*/MPa·m^1/2^	*a*_0_/mm	*B*/mm	(*W* − *a*_0_)/mm	*K_f_*/MPa·m^1/2^
1	5.827	10.17	12.35	0.33	31.426
2	6.072	10.21	12.47	0.35	33.325
3	6.181	11.08	12.61	0.36	35.217

## References

[1] Verma J., Taiwade R.V. (2017). Effect of welding processes and conditions on the microstructure, mechanical properties and corrosion resistance of duplex stainless steel weldments—A review. J. Manuf. Process..

[2] Magnabosco I., Ferro P., Bonollo F., Arnberg L. (2006). An investigation of fusion zone microstructures in electron beam welding of copper–stainless steel. Mater. Sci. Eng. A.

[3] Chung F.K., Wei P.S. (1999). Mass, Momentum, and Energy Transport in a Molten Pool When Welding Dissimilar Metals. J. Heat Transf..

[4] Sun Z., Karppi R. (1996). The application of electron beam welding for the joining of dissimilar metals: An overview. J. Mater. Process. Technol..

[5] Zumelzu E., Cabezas C. (1996). Study on welding such dissimilar materials as AISI 304 stainless steel and DHP copper in a sea-water environment. Influence of weld metals on corrosion. J. Mater. Process. Technol..

[6] Mai T.A., Spowage A.C. (2004). Characterisation of dissimilar joints in laser welding of steel-kovar, copper-steel and copper-aluminium. Mater. Sci. Eng. A.

[7] Srinivasan P.B., Muthupandi V., Dietzel W., Sivan V. (2006). An assessment of impact strength and corrosion behaviour of shielded metal arc welded dissimilar weldments between UNS 31803 and IS 2062 steels. Mater. Des..

[8] Wei P.S., Kuo Y.K., Ku J.S. (2000). Fusion Zone Shapes in Electron-Beam Welding Dissimilar Metals. J. Heat Transf..

[9] Tosto S., Nenci F., Jiandong H. (2003). Microstructure of copper-AISI type 304L electron beam welded alloy. Mater. Sci. Technol..

[10] Weigl M., Schmidt M. Modulated laser spot welding of dissimilar copper-aluminium connections. Proceedings of the 6th International Conference on Multi-Material Micro Manufacture.

[11] Velu M., Bhat S. (2013). Metallurgical and mechanical examinations of steel-copper joints arc welded using bronze and nickel-base superalloy filler materials. Mater. Des..

[12] Wu M.F., Si N.C., Chen J. (2011). Contact reactive brazing of Al alloy/Cu/stainless steel joints and dissolution behaviors of interlayer. Trans. Nonferrous Met. Soc. China.

[13] Yaghi A.H., Hyde T.H., Becker A.A., Sun W. (2013). Finite element simulation of residual stresses induced by the dissimilar welding of a P92 steel pipe with weld metal IN625. Int. J. Press. Vessel. Pip..

[14] Yao C.W., Xu B.S., Zhang X.C., Huang J., Fu J., Wu Y.X. (2009). Interface microstructure and mechanical properties of laser welding copper-steel dissimilar joint. Opt. Lasers Eng..

[15] Zhang B.G., Zhao J., Li X.P., Chen G.Q. (2015). Effects of filler wire on residual stress in electron beam welded QCr0.8 copper alloy to 304 stainless steel joints. Appl. Therm. Eng..

[16] Liu S., Liu F., Xu C., Zhang H. (2013). Experimental investigation on arc characteristic and droplet transfer in CO_2_ laser–metal arc gas (MAG) hybrid welding. Int. J. Heat Mass Transf..

[17] Liu F., Zhang Z., Liu L. (2012). Microstructure evolution of Al/Mg butt joints welded by gas tungsten arc with Zn filler metal. Mater. Character..

[18] Dong H., Hu W., Duan Y., Wang X., Dong C. (2012). Dissimilar metal joining of aluminum alloy to galvanized steel with Al–Si, Al–Cu, Al–Si–Cu and Zn–Al filler wires. J. Mater. Process. Technol..

[19] Chen S.H., Li L.Q., Chen Y.B., Liu D.J. (2010). Si diffusion behavior during laser welding-brazing of Al alloy and Ti alloy with Al-12Si filler wire. Trans. Nonferrous Met. Soc. China.

[20] Li H.M., Sun D.Q., Cai X.L., Dong P., Wang W.Q. (2012). Laser welding of TiNi shape memory alloy and stainless steel using Ni interlayer. Mater. Des..

[21] Miles M., Kohkonen K., Weickum B., Feng Z. (2009). Friction Bit Joining of Dissimilar Material Combinations of High Strength Steel DP 980 and Al Alloy AA 5754. SAE Tech. Pap..

[22] Curtis T., Widener C., West M., Jasthi B., Hovanski Y., Carlson B., Szymanski R., Bane W., Mishra R.S., Mahoney M.W., Sato Y., Hovanski Y. (2015). Friction Stir Scribe Welding of Dissimilar Aluminum to Steel Lap Joints. Friction Stir Welding and Processing VIII.

[23] Semak V., Matsunawa A. (1999). The role of recoil pressure in energy balance during laser materials processing. J. Phys. D Appl. Phys..

[24] IMAM How Do SLM Process Defects Impact Ti64 Mechanical Properties?. http://www.insidemetaladditivemanufacturing.com/blog/how-doslm-process-defects-impact-ti64-mechanical-properties.

[25] Kar J., Roy S.K., Roy G.G. (2016). Effect of beam oscillation on electron beam welding of copper with AISI-304 stainless steel. J. Mater. Process. Technol..

[26] Wei P.S., Chung F.K. (2000). Unsteady Marangoni Flow in a Molten Pool When Welding Dissimilar Metals. Metall. Mater. Trans. B.

[27] Blouin A., Chapuliot S., Marie S., Niclaeys C., Bergheau J.M. (2014). Brittle fracture analysis of Dissimilar Metal Welds. Eng. Fract. Mech..

[28] Gilles P., Brosse A., Pignol M. Simulation of Ductile Tearing in a Dissimilar Material Weld up to Pipe Wall Break-Through. Proceedings of the Asme Pressure Vessels & Piping Division/k-pvp Conference.

[29] Samal M.K., Seidenfuss M., Roos E., Balani K. (2011). Investigation of failure behavior of ferritic–austenitic type of dissimilar steel welded joints. Eng. Fail. Anal..

[30] (2014). GB/T 21143-2014 Metallic Materials-Unified Method of Test for Determination of Quasistatic Fracture Toughness.

[31] Faidy C. Structural Integrity of Bi-Metallic Welds in Piping Fracture Testing and Analysis. Proceedings of the Asme Pressure Vessels & Piping Conference.

[32] Ashari S.E., Mohammadi S. (2011). Delamination analysis of composites by new orthotropic bimaterial extended finite element method. Int. J. Numer. Methods Eng..

[33] Belytschko T.Y., Black T. (2015). Elastic Crack Growth in Finite Elements with Minimal Remeshing. Int. J. Numer. Methods Eng..

[34] Nicak T., Schendzielorz H., Keim E., Meier G. STYLE: Study on Transferability of Fracture Material Properties from Small Scale Specimens to a Real Component. Proceedings of the Asme Pressure Vessels & Piping Conference.

[35] Motamedi D., Mohammadi S. (2009). Dynamic crack propagation analysis of orthotropic media by the extended finite element method. Int. J. Fract..

[36] Zhang Z., Ma W.L., Wu H.L., Wu H.P., Jiang S.F., Chai G.Z. (2018). A rigid thick Miura-Ori structure driven by bistable carbon fibre-reinforced polymer cylindrical shell. Compos. Sci. Technol..

[37] Zhang Z., Li Y., Wu H.L., Chen D.D., Yang J., Wu H.P., Jiang S.F., Chai G.Z. (2018). Viscoelastic bistable behaviour of antisymmetric laminated composite shells with time-temperature dependent properties. Thin-Walled Struct..

[38] Rivalin F., Besson J., Pineau A., Fant M.D. (2001). Ductile tearing of pipeline-steel wide plates: II. Modeling of in-plane crack propagation. Eng. Fract. Mech..

[39] Kar J., Dinda S.K., Roy G.G., Roy S.K., Srirangam P. (2018). X-ray tomography study on porosity in electron beam welded dissimilar copper–304SS joints. Vacuum.

[40] Guo S., Zhou Q., Kong J., Peng Y., Xiang Y., Luo T., Wang K., Zhu J. (2016). Effect of beam offset on the characteristics of copper/304stainless steel electron beam welding. Vacuum.

[41] Zhang B.G., Zhao J., Xiao-Peng L.I., Feng J.C. (2014). Electron beam welding of 304 stainless steel to QCr0.8 copper alloy with copper filler wire. Trans. Nonferrous Met. Soc. China.

[42] Chen G., Shu X., Liu J., Zhang B., Feng J. (2020). Crystallographic texture and mechanical properties by electron beam freeform fabrication of copper/steel gradient composite materials. Vacuum.

[43] Tomashchuk I., Sallamand P., Jouvard J.M., Grevey D. (2010). The simulation of morphology of dissimilar copper–steel electron beam welds using level set method. Comput. Mater. Sci..

[44] (2012). GB/T 5231-2012 Designation and Chemical Composition of Wrought Copper and Copper Alloys.

[45] (2015). GB/T 699-2015 Quality Carbon Structure Steels.

[46] (2010). GB/T 228.1-2010 Metallic Materials-Tensile Testing-Part 1: Method of Test at Room Temperature.

[47] (2012). GB/T 28896-2012 Metallic Materials-Method of Test for the Determination of Quasistatic Fracture Toughness of Welds.

[48] Turichin G.A., Klimova O.G., Babkin K.D., Pevzner Y.B. (2014). Effect of Thermal and Diffusion Processes on Formation of the Structure of Weld Metal in Laser Welding of Dissimilar Materials. Met. Sci. Heat Treat..

[49] Qin E.W., Lu L., Tao N.R., Tan J., Lu K. (2009). Enhanced fracture toughness and strength in bulk nanocrystalline Cu with nanoscale twin bundles. Acta Mater..

